# Behavioral Response, Fumigation Activity, and Contact Activity of Plant Essential Oils Against Tobacco Beetle (*Lasioderma serricorne* (F.)) Adults

**DOI:** 10.3389/fchem.2022.880608

**Published:** 2022-03-24

**Authors:** Yanling Ren, Tao Wang, Yingjie Jiang, Ding Chen, Wenyu Zuo, Jianjun Guo, Daochao Jin

**Affiliations:** ^1^ Guizhou Provincial Key Laboratory for Agricultural Pest Management of the Mountainous Region, Institute of Entomology, Scientific Observing and Experimental Station of Crop Pest in Guiyang, Ministry of Agriculture, Guizhou University, Guiyang, China; ^2^ Guizhou Light Industry Technical College, Guiyang, China; ^3^ China Tobacco Guizhou Import and Export Co., Ltd., Guiyang, China; ^4^ Guizhou Tobacco Redrying Co., Ltd., Guiyang Redrying Factory, Guiyang, China

**Keywords:** plant essential oils, behavioral response, fumigation activity, contact activity, *Lasioderma serricorne* (F.)

## Abstract

Tobacco beetle (*Lasioderma serricorne* (F.)) is one of the main storage pests that harm tobacco leaves. The current control methods mainly include physical control, chemical control, and biological control, but they all have their own disadvantages. In this study, 22 kinds of plant essential oils in grapefruit, peppermint, juniper, eucalyptus, myrrh, lemon grass, geranium, tea tree, cypress, citronella, patchouli, benzoin, rosemary, cinnamon, clary sage, bergamot, mastic, ginger, rose hydrosol, cedar, thyme, and basil, respectively, are selected to explore their behavioral responses against *L. serricorne* adults using a glass Y-tube olfactometer. The behavioral responses results show that 17 kinds of essential oils in eucalyptus, basil, grapefruit, cypress, mastic, peppermint, patchouli, juniper, geranium, thyme, benzoin, lemon grass, cinnamon, ginger, rosemary, clary sage, and citronella can avoid *L. serricorne* adults, while five kinds of essential oils in tea tree, rose hydrosol, myrrh, bergamot, and cedar can attract *L. serricorne* adults. Especially, essential oils in eucalyptus and grapefruit can avoid *L. serricorne* adults at 1 μl/L with the repellent rates of 94.67 and 94.56%, respectively. Meanwhile, 17 kinds of essential oils which can avoid *L. serricorne* adults are selected to determine their fumigation activity against *L. serricorne* adults using the Erlenmeyer flask test method, and bioassay results show that after 72 h of treatment, five kinds of plant essential oils in rosemary, eucalyptus, basil, citronella, and geranium show excellent fumigation activity against *L. serricorne* adults with the mortality rates of 100.00, 95.29, 95.29, 94.12, and 91.76%, respectively, and their LD_50_ of the contact activity against *L. serricorne* adults determined using the leaf-dipping method are 3.60, 3.49, 8.90, 6.70, and 7.80 μl/L, respectively. Our results show that plant essential oils could be developed as environmentally friendly insect control agents.

## Introduction

Cigarette beetle (*Lasioderma serricorne* (F.)), a worldwide storage pest, caused harm to stored goods in China, United States, India, and other countries (Shaymaa et al., 2019). The control of *L. serricorne* is great significance to reduce the loss rate of stored goods. At present, methods such as control atmosphere (Cao et al., 2015; Chaitanyam et al., 2017; Kumar et al., 2017; Xu et al., 2017; Sun et al., 2020), control temperature (Makhijani and Gurney, 1995; Yu et al., 2011; Li et al., 2018), and installation of barrier nets (Chen et al., 2013) in physical control methods, phosphine fumigation (Peng et al., 2015; Fukazawa and Takahashi, 2017; Wu et al., 2017), and pesticide methods (Xiong et al., 2014; Tang et al., 2015; Li et al., 2021) in chemical control methods, and natural enemies (such as *Beauveria bassiana* and *Anisopteromalus calandrae*) in biological control methods (Kaelin et al., 1994; Kaelin et al., 1999; Guo et al., 2021; Khanum and Javed, 2021) are more popular control methods. However, physical control is more effective inside the warehouse but has little effect on the *L. serricorne* outside the warehouse; the current application range and types of biological control are not extensive; chemical control methods will inevitably produce residues and lead to *L. serricorne* resistance (Rajendran and Narasimhan, 1994; Zettler and Keevr, 1994; Savvidou et al., 2003; Silva et al., 2017). Therefore, the development of novel and eco-friendly control agents and methods is essential for the control of *L. serricorne*.

Over the past few decades, the world has been studying to find alternatives to biological control, especially plant essential oils. At present, the use of plant extracts has made significant progress in the prevention and control of pests. Since the 1980s, research on plant essential oils against *L. serricorne*, such as the lure or avoidance (Işikber et al., 2009; Guarino et al., 2021), fumigation activity (Işikber et al., 2009; Boukaew et al., 2017), contact activity (Huang and Ho, 1998; Huang et al., 2002; Naveen et al., 2021), has been carried out. Ramadan et al. (2020) studied the avoidance of *L. serricorne* by carvacrol, citronella, geraniol, nootkatone, and *N*,*N*-diethyl-meta-toluamide. Kamal et al. (2019) studied the avoidance of *L. serricorne* by extracts of sponge gourd (*Luffa aegyptiaca*), ajwain/caraway seeds (*Carum copticum*), and turmeric (*Curcuma longa*). The lure effect of *Capsicum* spp. dried fruit odorants against *L. serricorne* was studied by Guarino et al., which showed that *Capsicum annuum* and *Capsicum frutescens* have an attractive effect on *L. serricorne* (Guarino et al., 2021). In 2016, Lü and Liu found that the citronellal and citral had attractive activities against *L. serricorne* at a low concentration and had repellent activity against *L. serricorne* at higher concentration. Meanwhile, some reported literatures also showed that some plant essential oils, such as *Anethum graveolens*, *Azadirachta indica*, *Eucalyptus globulus*, *Mentha piperita*, and *Artemisia dubia*, revealed good fumigation and contact activity against *L. serricorne* (Khemira et al., 2012; Karakoc et al., 2018; Cheng et al., 2019; Naveena et al., 2021; Yang et al., 2021)*.*

In this study, 22 kinds of plant essential oils were selected: grapefruit, peppermint, juniper, eucalyptus, myrrh, lemon grass, geranium, tea tree, cypress, citronella, patchouli, benzoin, rosemary, cinnamon, clary sage, bergamot, mastic, ginger, rose hydrosol, cedar, thyme, and basil, respectively, and for the first time their behavioral response, fumigation activity, and contact activity against *L. serricorne* adults was studied.

## Material and Methods

### Insect Collection and Rearing

Samples (*L. serricorne*) were collected from the Guizhou Tobacco Redrying Co., Ltd., Guiyang Redrying Factory, and then placed in the Department of Guizhou Light Industrial Technical College for breeding with corn:tobacco foam:beer yeast = 90:5:5 as food. After that, *L. serricorne* were raised in an artificial intelligence climate box (LAC-450HPY-2, Shanghai Longyue Co., Ltd.) at a temperature, relative humidity, and photoperiod of 25 ± 1°C, 75 ± 5%, and 14L: 10D, respectively.

### Behavioral Response Test

The behavioral responses of 22 kinds of plant essential oils (99% purity), provided by Beijing Maosi Trading Company (Beijing, China), against *L. serricorne* adults were determined using a glass Y-tube olfactometer (Yancheng Xinmingte Glass Instrument Co., Ltd., Yancheng, China) (Li et al., 2014). Each 1 μl plant essential oil was dripped in a 1 L pre-washed bottle and acetone (1 μl) served as the negative control. After turning on the air pump for 5 min, 50 two-day-old *L. serricorne* adults pre-starved for 8 h were placed in the middle of the straight arm of the Y-type olfactometer. Three replicates were conducted for each treatment. After 5 min of treatment, the repellent rate of each plant essential oil is calculated using the following formula, where *N*_*c*_ represents the number of insects in the blank arm and *N*_*t*_ represents the number of insects in the treatment arm.

$$Repellent\;rate\;\left(\%\right)=\frac{N_{c}\;-\;N_{t}}{N_{c}\;+\;N_{t}}\times \;100.$$

### Fumigant Activity Test

The fumigation activities against *L. serricorne* adults of 17 kinds of essential oils which can avoid *L. serricorne* adults were studied using the Erlenmeyer flask test method (Wu et al., 2015). Each plant essential oil (15 μl) was dripped into a rectangular filter paper (1.5 cm × 4.0 cm), then the filter paper was hung vertically in the middle of a 1 L pre-washed bottle which contained 10 g Flue-cured tobacco leaves (Yunyan 85) and 30 two-day-old *L. serricorne* adults inside. Acetone (15 μl) served as the negative control. Each treatment was conducted three times. After 48 and 72 h of treatment, the mortality rate is determined, and the corrected mortality rate is calculated using the Abbott’s formula.

$$Mortality\;rate\;\left(\%\right)=\frac{Number\;of\;dead\;in\sec ts}{Number\;of\;test\;in\sec ts}\times 100,$$

$$Corrected\;mortality\;rate\;\left(\%\right)=\frac{Mortality\;rate\;of\;treatment\;group-Mortality\;rate\;of\;control\;group}{1-\;Mortality\;rate\;of\;control\;group}\times 100.$$

### Contact Activity Test

The plant essential oils with good fumigation activity were selected to study their contact activity against *L. serricorne* adults using the leaf-dipping method (Yuan et al., 2018). Five concentration gradients of each essential oil were diluted with acetone (200 ml). Flue-cured tobacco leaves (Yunyan 85) with the same growth condition were dipped into each concentration gradients of each essential oil for 30 s, and then dried in the air. After that, the Flue-cured tobacco leaves (Yunyan 85) were placed in a box (19.5 cm × 13.4 cm × 4.0 cm). 20 two-day-old *L. serricorne* adults were transferred to the box. Acetone served as the negative control, Pirimiphos-methyl (Actellic 50 EC^®^, Syngenta AG, Cape Town, South Africa) and Chlorantraniliprole (Zhengzhou Salongda Weixin Pesticide Co., Ltd., Henan, China) were selected as positive controls according to the research studies reported by Wang et al. (2011) and Han et al. (2014). Three replicates were conducted for each treatment. After 72 h of treatment, the mortality rate is determined and corrected using the Abbott’s formula.

### Statistical Analysis

All data represented in this study are analyzed using SPSS version 23 software (IBM, NY, United States). The toxic regression equation and LD_50_ values are analyzed by the Probit model from SPSS. The *p* value lower than 0.05, analyzed by statistical significance, is considered to be significant.

## Results and Discussion

### Behavioral Response

**FIGURE 1:**
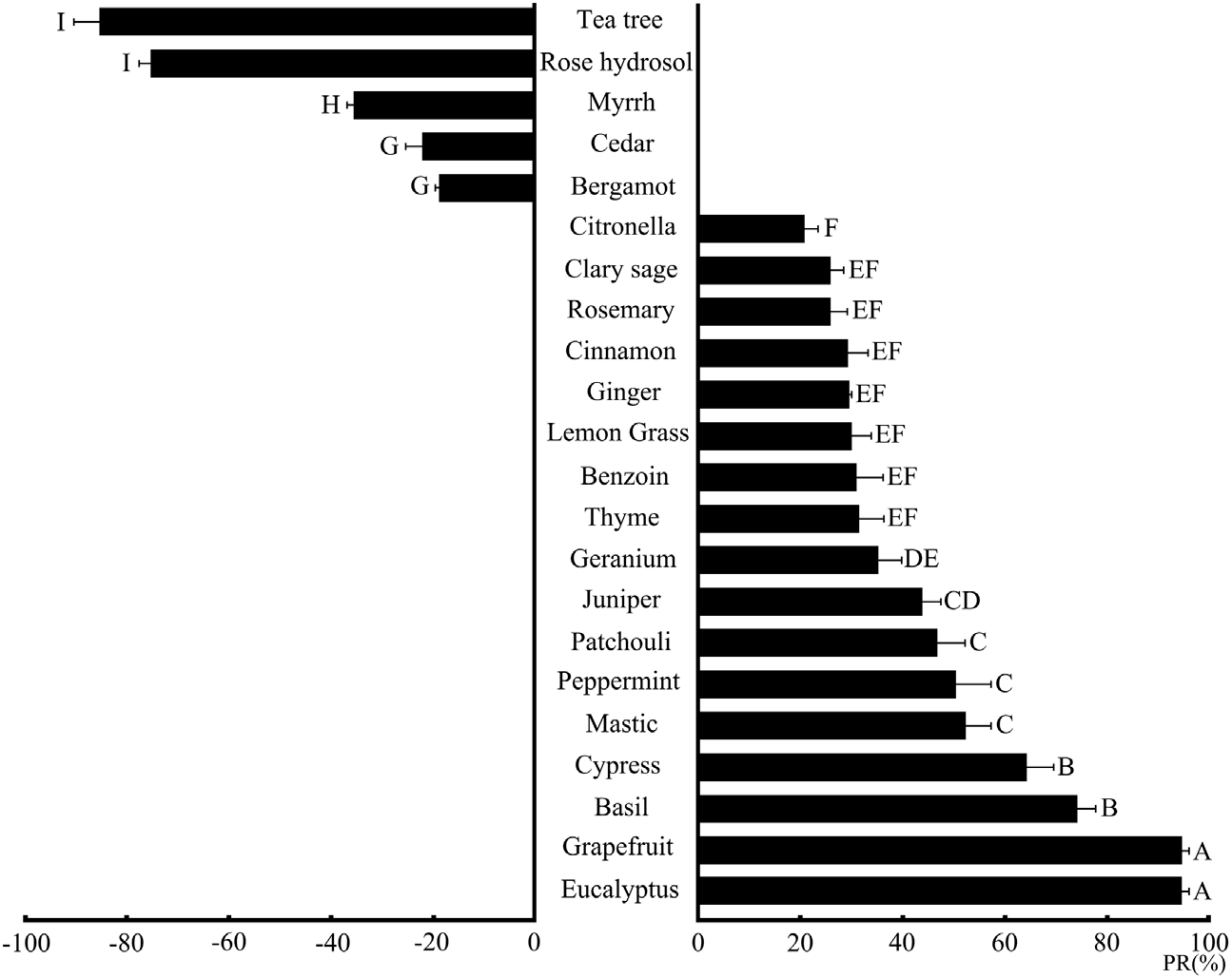
Behavioral response of 22 plant essential oils against *L. serricorne* adults. Different uppercase letters represented in the figure indicate a significant difference through LSD among different plant essential oils against *L. serricorne* adults. PR (%), repellent rate (Mean ± SE).

In this study, a total of 22 kinds of plant essential oils are selected to explore their behavioral responses against *L. serricorne* adults. Our results (Figure 1) show that 17 kinds of plant essential oils in eucalyptus, basil, grapefruit, cypress, mastic, peppermint, patchouli, juniper, geranium, thyme, benzoin, lemon grass, cinnamon, ginger, rosemary, clary sage, and citronella can avoid *L. serricorne* adults with the repellent rates of 20.79–94.67%. Especially, essential oils in eucalyptus and grapefruit are found to be the most successful plant essential oils that caused the maximum repellent rate (94.67 and 94.56%, respectively) against *L. serricorne* adults over the whole exposure period followed by basil (74.15%) and cypress (64.31%). Similar results reported by Song et al. (2018) showed that eucalyptus essential oil (500 μl) can avoid *L. serricorne* adults up to 67%, whereas Tampe et al. (2020) reported that eucalyptus essential oil (500 ng) was attractive for both sexes of *Aegorhinus superciliosus*. Meanwhile, Figure 1 also shows that five kinds of essential oils in tea tree, myrrh, bergamot, rose hydrosol, and cedar can attract *L. serricorne* adults with the repellent rates of −18.56– −84.98%; among them, the tea tree essential oil has the most attractive effect on *L. serricorne* with a repellent rate of −84.98% followed by rose hydrosol (−74.85%). Buteler et al. (2019) identified the behaviour effect of tea tree essential oil on *Acromyrmex* spp. ants, and the results showed that the tea tree essential oil (10 ml/L) can 69% avoid *Acromyrmex* spp. Ants at 30 min. Diaz–Montano and Trumble (2013) reported that tea tree essential oil (2000 μl) showed a significant repellency on potato psyllid (*Bactericera cockerelli*) adults.

### Fumigant Activity

Base on the behavioral responses of the plant essential oils against *L. serricorne* adults, 17 kinds of plant essential oils, which can avoid *L. serricorne* adults, are selected to study their fumigation activity against *L. serricorne* adults at 15 μl/L. Table 1 shows that, after 48 and 72 h of treatment, some of the plant essential oils exhibit good fumigation activity against *L. serricorne* adults at 15 μl/L. Among of them, after 72 h of treatment, rosemary essential oil shows the best fumigation activity against *L. serricorne* adults with a 100.00% mortality rate. Yang et al. (2020) selected 28 kinds of essential oils to evaluate their fumigation activity against maize weevils (*Sitophilus zeamais*), the results showed that essential oils in cinnamon (LD_50_ = 0.04 mg/cm^2^), ylang ylang (LD_50_ = 0.032 mg/cm^2^), and tea tree (LD_50_ = 0.15 mg/cm^2^) revealed superior fumigation activity against maize weevils. Meanwhile, Trivedi et al. (2017) reported that the LD_50_ values of fumigation activity obtained for 24, 48 and 72 h for rosemary essential oil against the stored grain pest *Callosobruchus chinensis* were 3.282, 4.261, and 1.509 mg/L, respectively. In addition, Çetin and Güdek (2020) found that rosemary essential oil exhibited perfect fumigation activity against fifth instar larvae of the date moth *Ectomyelois ceratoniae* with the mortality rate of 100.00% at 90 μl/L after 30 days of exposure.

**TABLE 1:** Fumigation activity of 17 kinds of plant essential oils against *L. serricorne* adults at 15 μl/L.

Essential oil	Fumigation activity (mean ± SE) (%)^a^	
	48 h	72 h
Rosemary	40.00 ± 1.92 C	100.00 A
Eucalyptus	53.33 ± 1.92 B	95.29 ± 3.11 A
Basil	25.56 ± 1.11 D	95.29 ± 11.77 A
Citronella	74.44 ± 4.84 A	94.12 ± 2.35 A
Geranium	77.78 ± 2.94 A	91.76 ± 31.13 A
Thyme	40.00 ± 3.85 C	76.47 ± 3.11 B
Clary sage	27.78 ± 1.11 D	71.76 ± 4.08 B
Cinnamon	31.11 ± 1.11 D	51.76 ± 1.18 C
Juniper	11.11 ± 1.11 E	47.06 ± 5.39 C
Grapefruit	8.89 ± 1.11 EF	36.47 ± 3.53 D
Ginger	11.11 ± 1.11 E	22.35 ± 2.04 E
Patchouli	0 G	21.17 ± 2.35 E
Peppermint	0 G	18.82 ± 2.03 E
Benzoin	0 G	9.41 ± 2.35 F
Lemon grass	3.33 ± 1.92 FG	7.05 ± 1.18 F
Mastic	11.11 ± 1.11 E	5.88 ± 1.18 F
Cypress	0 G	5.88 ± 1.18 F

a Different uppercase letters indicate the fumigation activity against *L. serricorne* adults of the plant essential oils with a significant difference through LSD.

### Contact Activity

Five kinds of plant essential oils, rosemary, eucalyptus, basil, citronella, and geranium, are selected to study their contact activity against *L. serricorne* adults. Table 2 shows that, after 72 h of treatment, plant essential oils in rosemary, eucalyptus, basil, citronella, and geranium exhibit good contact activity against *L. serricorne* adults, with the mortality rates of 5.35%–100.00%, 7.14%–100.00%, 10.71%–89.29%, 7.14%–92.86%, and 12.5%–94.64%, respectively. Especially, two kinds of plant essential oils in rosemary and eucalyptus revealed a 100% mortality rate against *L. serricorne* adults at 10 and 15 μl/L, respectively. Meanwhile, Table 3 shows that the LD_50_ of the contact activity against *L. serricorne* adults of plant essential oils in rosemary, geranium, citronella, basil, and eucalyptus are 3.60, 3.49, 8.90, 6.70, and 7.80 μl/L, respectively, which are even better than those of Pirimiphos-methyl (15.45 μl/L) and Chlorantraniliprole (249.77 μl/L). In recent years, many research studies on the essential oils against *L. serricorne* have been performed, for example, Liang et al. (2021) found that *Elsholtzia densa* essential oil possesses obvious contact activity (LD_50_ = 24.29 mg/L) against *L. serricorne*. Meanwhile, Zhou et al. (2018) reported that the *Artemisia lavandulaefolia* (Compositae) essential oil also exhibited good contact toxicity (LD_50_ = 13.51 μg/L) to control *L. serricorne*.

**TABLE 2:** Contact activity of five kinds of plant essential oils against *L. serricorne* adults.

Essential oil	Contact activity (mean ± SE) (%)^a^							
	0.5 μl/L	1 μl/L	2.5 μl/L	5 μl/L	10 μl/L	15 μl/L	20 μl/L	40 μl/L
Rosemary	5.35 ± 1.79 E	10.71 ± 1.79 D	39.28 ± 1.79 C	69.64 ± 1.79 B	100.00 A	-	-	-
Eucalyptus	7.14 ± 3.57 E	42.86 ± 1.79 D	-	71.43 ± 1.79 C	89.29 ± 0.03 B	100.00 A	-	-
Basil	-	10.71 ± 1.79 D	-	41.07 ± 3.09 C	51.78 ± 6.19 B	80.36 ± 1.79 A	89.29 ± 0.01 A	-
Citronella	-	7.14 ± 1.79 D	-	48.21 ± 3.57 C	58.93 ± 3.57 C	-	73.21 ± 5.36 B	92.86 ± 1.79 A
Geranium	-	12.5 ± 3.57 E	-	41.07 ± 3.09 D	64.28 ± 1.79 C	82.14 ± 3.57 B	94.64 ± 3.09 A	-

a Different uppercase letters indicate the contact activity against *L. serricorne* adults of the plant essential oils with a significant difference through LSD.

**TABLE 3:** The LD_50_ values of the contact activity against *L. serricorne* adults of the tested plant essential oils.

Treatment	Toxic regression equation	Chi-Square	LD_50_ (mean ±95% confidence limit) (μl/L)
Rosemary	y = -1.639 + 0.456x	0.23	3.60 (1.26–83.63)
Eucalyptus	y = -0.812 + 0.233x	1.04	3.49 (-10.18–14.68)
Basil	y = -1.094 + 0.123x	0.39	8.90 (-16.40–22.12)
Citronella	y = -1.372 + 0.721x	0.36	6.70 (0.01–35.58)
Geranium	y = -1.072 + 0.138x	0.39	7.80 (-12.20–16.82)
Pirimiphos-methyl	y = -0.651 + 0.042x	3.15	15.45 (-7.05–34.27)
Chlorantraniliprole	y = -2.033 + 0.003x	3.91	249.77 (305.02–689.48)

## Conclusion

In this study, 22 kinds of plant essential oils are selected to study their behavioral response, fumigant activity, and contact activity against *L. serricorne* adults. Our results show that five plant essential oils can attract *L. serricorne* adults, whereas 17 plant essential oils can avoid *L. serricorne* adults. Meanwhile, rosemary essential oil shows the best fumigation activity against *L. serricorne* adults, and eucalyptus essential oil shows the best contact activity against *L. serricorne* adults, supporting the interest of industrial use of plant essential oils, such as rosemary and geranium essential oils, as environmentally friendly insect control agents.

## Data Availability

The original contributions presented in the study are included in the article/Supplementary Material; further inquiries can be directed to the corresponding authors.
